# Inflammatory and Lipid Biomarkers in Early Atherosclerosis: A Comprehensive Analysis

**DOI:** 10.3390/life14101310

**Published:** 2024-10-16

**Authors:** Alim Namitokov, Karina Karabakhtsieva, Olga Malyarevskaya

**Affiliations:** 1 Department of Therapy #1, Kuban State Medical University, Sedina Street 4, 350063 Krasnodar, Russia; 2 Scientific Research Institute, Regional Clinical Hospital #1 NA Prof. S.V. Ochapovsky, 1st May Street 167, 350086 Krasnodar, Russia; karina26051998@gmail.com (K.K.);

**Keywords:** atherosclerosis, inflammatory indices, lipid biomarkers, SIRI, ANGPTL3, young adults, machine learning, personalized medicine, cardiovascular risk, early detection

## Abstract

Introduction: Atherosclerosis is a leading cause of cardiovascular disease, characterized by lipid accumulation and chronic inflammation within arterial walls. Early detection in young adults is crucial for preventing adverse cardiovascular events. This study investigates the associations between inflammatory indices, lipid biomarkers, and the presence of atherosclerosis in patients aged 18 to 55 years. Methods: A cross-sectional study was conducted involving 89 participants divided into two groups: 62 patients with documented atherosclerosis (main group) and 27 healthy controls without significant atherosclerosis. Comprehensive data—including demographic information, medication use, imaging results, laboratory parameters, and calculated inflammatory indices (SIRI, SII, AISI, NLR, PLR, MLR)—were collected. Statistical analyses included correlation assessments, group comparisons using the Mann–Whitney U test, logistic regression modeling, feature importance analysis with Random Forest and Gradient Boosting classifiers, receiver operating characteristic (ROC) curves, and K-means clustering. Results: Significant differences were observed between the main and control groups. Patients with atherosclerosis exhibited elevated inflammatory indices (SIRI, NLR, MLR, SII) and lipid profile abnormalities (higher TC and LDL-C, lower HDL-C). Lp(a) and ANGPTL3 levels were significantly higher in the main group (*p* < 0.001 and *p* < 0.01, respectively). Logistic regression identified SIRI and ANGPTL3 as significant predictors of atherosclerosis, with the model demonstrating high accuracy (77%) and sensitivity (93%). Feature importance analysis confirmed the significance of SIRI and ANGPTL3, alongside traditional lipid biomarkers, in predicting disease presence. ROC analysis showed excellent model performance (AUC > 0.80). Clustering analysis revealed two distinct patient subgroups characterized by predominant inflammatory profiles or lipid metabolism disturbances. Conclusions: Systemic inflammation and lipid abnormalities play significant roles in early atherosclerosis among young adults. Elevated SIRI and ANGPTL3 levels are potent predictors of disease presence. The integration of inflammatory indices and lipid biomarkers into predictive models enhances risk stratification and supports personalized medicine approaches.

## 1. Introduction

Atherosclerosis is a chronic inflammatory disease characterized by the accumulation of lipids, inflammatory cells, and fibrous elements within the arterial wall, leading to plaque formation and vascular obstruction. It is a leading cause of morbidity and mortality worldwide, significantly contributing to conditions such as coronary artery disease (CAD), stroke, and peripheral arterial disease (PAD) [1]. Despite advances in therapeutic interventions, early detection and prevention remain critical, especially in younger populations who may present with subclinical disease yet are at increased risk of future cardiovascular events.

Inflammation plays a pivotal role in the initiation and progression of atherosclerosis. Various inflammatory indices, such as the Systemic Inflammatory Response Index (SIRI), Systemic Inflammation Index (SII), Neutrophil–Lymphocyte Ratio (NLR), Platelet–Lymphocyte Ratio (PLR), and Monocyte–Lymphocyte Ratio (MLR), have emerged as potential biomarkers for cardiovascular risk assessment [2,3]. These indices reflect the balance between pro-inflammatory and anti-inflammatory cells, offering insights into the systemic inflammatory status of individuals and their potential risk for atherogenesis.

In addition to inflammation, lipid abnormalities are central to the pathogenesis of atherosclerosis. Lipid-related biomarkers like Lipoprotein(a) [Lp(a)], Angiopoietin-like protein 3 (ANGPTL3), Lipoprotein-associated Phospholipase A2 (Lp-PLA2), and Apolipoprotein B (ApoB) have been implicated in the development and progression of atherosclerotic plaques [4,5]. Elevated levels of low-density lipoprotein cholesterol (LDL-C) and total cholesterol (TC) are well-established risk factors, while high-density lipoprotein cholesterol (HDL-C) is considered protective [6].

Understanding the interplay between inflammatory markers and lipid biomarkers is crucial for identifying individuals at high risk of developing atherosclerosis. This study aims to explore these relationships in a young patient cohort, providing insights that could contribute to targeted prevention strategies and personalized medicine approaches.

## 2. Materials and Methods

We conducted a cross-sectional study involving 89 patients aged between 18 and 55 years. The inclusion criteria were patients who were stable without signs of acute coronary syndrome and had no severe chronic diseases, systemic, or rheumatological conditions. All participants had preserved left ventricular ejection fraction and normal renal function. The study population was divided into two groups: the main group consisted of 62 patients with atherosclerosis detected in the coronary, carotid, or lower limb arteries (some exhibiting multiple localizations), while the control group included 27 healthy individuals without signs of hemodynamically significant atherosclerosis.

Inclusion and exclusion criteria were applied to ensure the appropriate selection of participants in accordance with WHO guidelines.

The inclusion criteria included the following:

1. Age: patients between 18 and 55 years old.
2. Health condition: patients with documented atherosclerosis in the coronary, carotid, or peripheral arteries, confirmed by invasive and non-invasive diagnostic methods.
3. No acute conditions: exclusion of patients showing signs of acute coronary syndrome.
4. Cardiac function: presence of preserved left ventricular ejection fraction (e.g., >50%).
5. General health: patients without severe chronic diseases such as chronic kidney failure, oncological, or rheumatological diseases that could affect lipid metabolism or the inflammatory process.

The exclusion criteria included the following:

1. Acute conditions: patients with signs of acute coronary syndrome or other severe cardiac pathology requiring urgent intervention.
2. Comorbid conditions: patients with severe systemic diseases (e.g., oncology, rheumatological, systemic inflammatory diseases, advanced kidney failure) that could distort study results.
3. Use of specific therapy: exclusion of patients who are taking medications that significantly affect lipid profiles or inflammation status, beyond standard therapy (e.g., investigational drugs such as PCSK-9 inhibitors).
4. Factors impacting data analysis: patients unable to comply with study requirements (e.g., cognitive impairments, non-compliance with medical advice).
5. Myocardial infarction in anamnesis.

Comprehensive data were collected for each participant, including demographic information (gender, smoking history, presence of diabetes and arterial hypertension, family history of cardiovascular diseases) and medication usage (statins, aspirin, P2Y12 inhibitors). All individuals included in the study are of the European (Caucasian) race. The main clinical and demographic characteristics of patients are presented in Table 1.

Imaging data provided a segment-by-segment description of the coronary arteries, indicating the degree of stenosis, as well as the maximum degree of stenosis in the carotid and peripheral arteries. Invasive angiography was used in all cases of diagnosis of atherosclerotic arterial lesions.

Laboratory parameters were obtained through blood tests, including complete blood counts (neutrophil, lymphocyte, monocyte, and platelet counts), creatinine, and lipid profiles TC, LDL-C, HDL-C, and TG. Specific atherosclerosis-related biomarkers were also measured, such as Lp(a), ANGPTL3, Lipoprotein-associated PhosLp-PLA2, and Apolipoprotein ApoB.

Inflammatory indices were calculated using the following formulas:

- SIRI: (Neutrophil count × Monocyte count) ÷ Lymphocyte count
- SII: (Neutrophil count × Platelet count) ÷ Lymphocyte count
- AISI: (Neutrophil count × Monocyte count × Platelet count) ÷ Lymphocyte count
- NLR: Neutrophil count ÷ Lymphocyte count
- PLR: Platelet count ÷ Lymphocyte count
- MLR: Monocyte count ÷ Lymphocyte count

Ethical approval was obtained from the institutional review board, and all participants provided informed consent before enrollment in the study.

Data were analyzed using Python (version 2.7.16) with relevant statistical libraries, including Pandas (version 2.0.3), NumPy (version 1.24.4), SciPy (version 1.10.1), scikit-learn (vesion 1.3.2), and Matplotlib (version 3.7.5). Continuous variables were tested for normality using the Shapiro–Wilk test, and due to non-normal distribution, non-parametric tests were employed. Outliers were identified using box plots and Z-scores, and log transformations were applied where necessary.

Pearson correlation coefficients were calculated to assess the relationships between variables within the main group. Group comparisons between the main and control groups were performed using the Mann–Whitney U test, and effect sizes were calculated using Cohen’s d. Logistic regression modeling was conducted to identify predictors of atherosclerosis, with the presence of atherosclerosis as the dependent variable and selected biomarkers and inflammatory indices as independent variables. Model performance was evaluated using accuracy, the confusion matrix, precision, recall, and the F1-score.

Feature importance analysis was carried out using Random Forest and Gradient Boosting classifiers to assess which biomarkers and indices had the strongest influence on the prediction of atherosclerosis. Receiver operating characteristic (ROC) curves were plotted, and the Area Under the Curve (AUC) was calculated to evaluate model performance. Optimal thresholds for key biomarkers were determined using Youden’s Index, which maximizes the difference between true positive and false positive rates. Clustering analysis using K-means clustering was applied to the dataset to identify distinct patient subgroups based on their biomarker profiles.

## 3. Results

### 3.1. Correlation Analysis

In the main group of patients with atherosclerosis, significant correlations were observed among various biomarkers and inflammatory indices.

**Figure 1:**
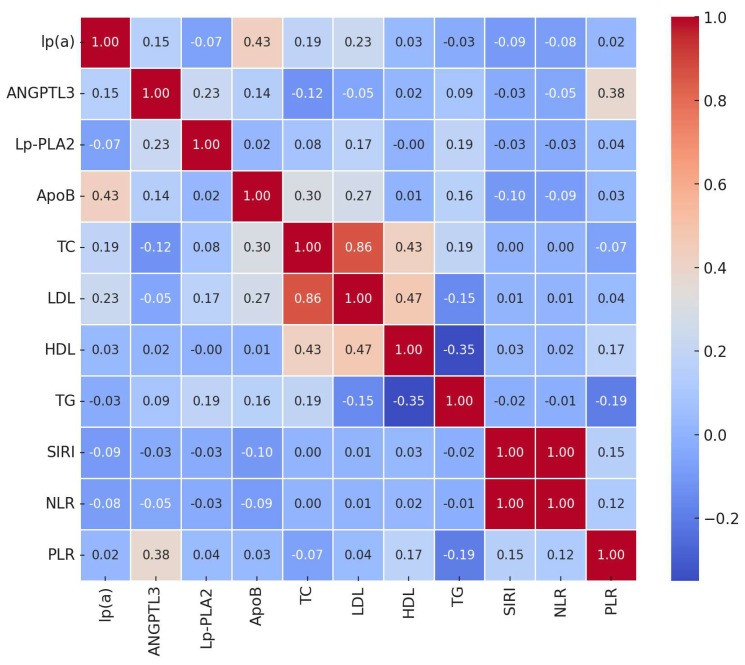
Correlation heatmap showing relationships among biomarkers and inflammatory indices in the main group.

Figure 1 illustrates the correlation heatmap of the main biomarkers.

Key findings include the following:

- Lp(a) showed a moderate positive correlation with ApoB (r = 0.43, *p* < 0.01), suggesting that higher levels of Lp(a) are associated with elevated ApoB levels.
- ANGPTL3 correlated positively with PLR (r = 0.38, *p* < 0.01), indicating a potential link between angiogenic factors and platelet-driven inflammation.
- Lp-PLA2 had a mild correlation with TG (r = 0.19, *p* = 0.05), consistent with its role in lipid metabolism.
- ApoB correlated with TC (r = 0.30, *p* < 0.01) and LDL-C (r = 0.27, *p* < 0.01), reflecting its involvement in the formation of atherogenic lipoprotein particles.

Interestingly, inflammatory indices such as SIRI, NLR, and PLR did not show strong correlations with most lipid biomarkers, suggesting that inflammation and lipid metabolism may contribute to atherosclerosis through independent but potentially synergistic pathways.

### 3.2. Group Comparisons

The Mann–Whitney U test revealed significant differences between the main and control groups.

Table 2 summarizes the median values and *p*-values for key variables.

These findings suggest that patients with atherosclerosis exhibit a heightened inflammatory state and lipid profile abnormalities compared to healthy controls.

### 3.3. Logistic Regression Modeling

A logistic regression model was developed to predict the presence of atherosclerosis based on selected biomarkers and inflammatory indices. The model demonstrated an accuracy of 77%. The confusion matrix showed that the model correctly predicted 13 out of 14 cases of atherosclerosis but misclassified some control group members as having the disease.

**Figure 2:**
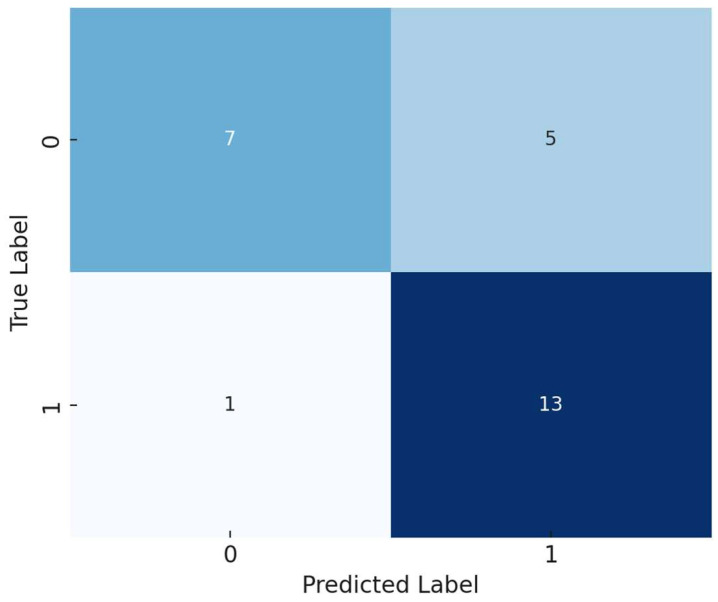
Confusion matrix of the logistic regression model.

Figure 2 shows the confusion matrix of the model.

- Precision (Positive Class): 0.72.
- Recall (Positive Class): 0.93.
- F1-Score (Positive Class): 0.81.

The model exhibited good sensitivity (ability to identify true positives) but moderate specificity (ability to identify true negatives).

### 3.4. Feature Importance Analysis

Feature importance analysis using Random Forest and Gradient Boosting classifiers identified the most significant predictors of atherosclerosis.

**Figure 3:**
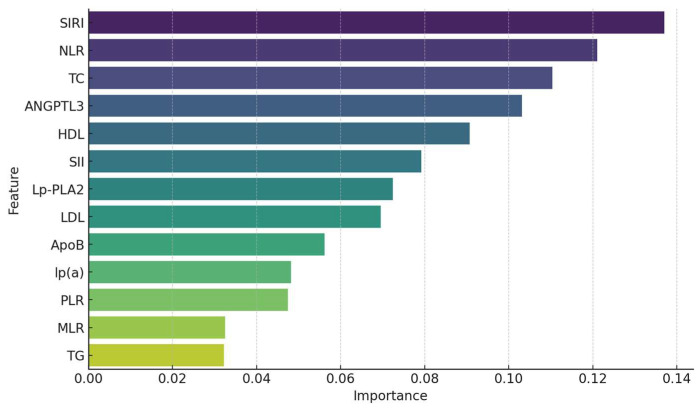
Feature importance scores from the Random Forest classifier.

Figure 3 presents the feature importance derived from the Random Forest model.

The top predictors of early atherosclerosis identified in our Random Forest feature importance analysis were SIRI, NLR, TC, ANGPTL3, and HDL-C. Notably, NLR ranked higher than LDL-C, which traditionally is considered a key marker of atherosclerosis. This highlights the potentially stronger role of inflammatory responses in this cohort, as reflected by NLR, in contrast to lipid-related factors like LDL-C.

These results underscore the importance of both inflammatory indices and lipid biomarkers in predicting early atherosclerotic disease.

### 3.5. ROC Curve and AUC

The ROC curves for the Random Forest and Gradient Boosting models demonstrated strong discriminatory ability.

**Figure 4:**
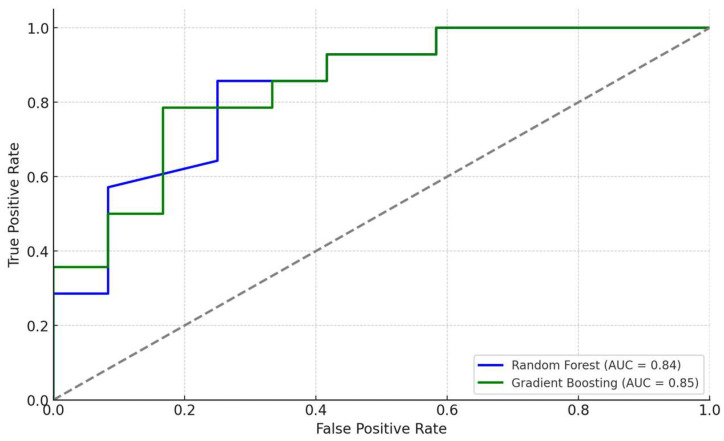
ROC curves for Random Forest and Gradient Boosting classifiers.

Figure 4 displays the ROC curves for both models.

- Random Forest AUC: 0.84.
- Gradient Boosting AUC: 0.85.

An AUC greater than 0.80 indicates excellent model performance in distinguishing between patients with and without atherosclerosis.

### 3.6. Optimal Thresholds

Using Youden’s Index, optimal thresholds for key biomarkers were determined.

Table 3 lists the thresholds along with sensitivity and specificity.

These thresholds may serve as clinical cut-off points for risk stratification.

### 3.7. Clustering Analysis

K-means clustering identified two distinct patient subgroups.

**Figure 5:**
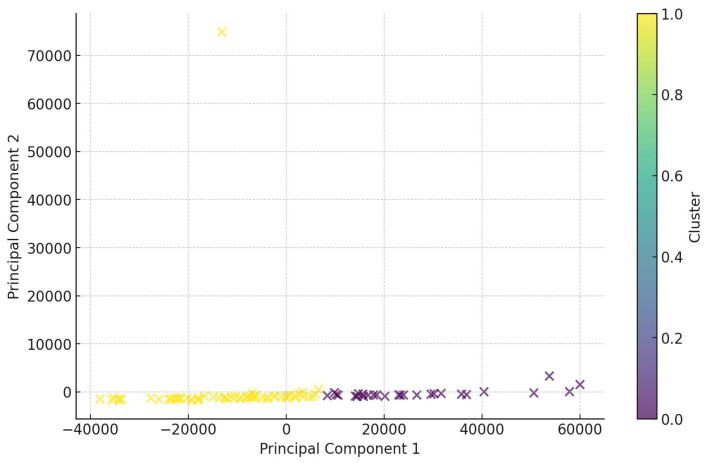
K-means clustering of patients showing two distinct clusters.

Figure 5 illustrates the clustering of patients based on biomarker profiles.

- Cluster 0 (*n* = 59): characterized by higher inflammatory indices (NLR, SII, SIRI).
- Cluster 1 (*n* = 27): exhibited higher levels of ANGPTL3 but lower inflammatory indices.

This suggests heterogeneity among patients with atherosclerosis, with some exhibiting a predominant inflammatory profile and others showing lipid metabolism disturbances as the primary feature.

## 4. Discussion

The present study provides a comprehensive analysis of the relationships between inflammatory indices, lipid biomarkers, and atherosclerosis in a young patient cohort. Our findings highlight the significant roles of both systemic inflammation and lipid abnormalities in the early stages of atherosclerotic disease.

### 4.1. Inflammatory Indices as Predictors of Atherosclerosis

Elevated inflammatory indices such as SIRI, NLR, MLR, and SII were observed in patients with atherosclerosis compared to healthy controls. SIRI emerged as the most significant predictor in both logistic regression and machine learning models. These indices reflect the balance between different leukocyte populations, which play critical roles in inflammation and immunity.

The NLR and MLR are indicators of neutrophil- and monocyte-mediated inflammatory responses, respectively. Neutrophils contribute to atherogenesis by releasing reactive oxygen species and proteolytic enzymes that damage the endothelium [7]. Monocytes migrate into the arterial wall, differentiate into macrophages, and take up oxidized LDL, forming foam cells that constitute the lipid-rich necrotic core of atherosclerotic plaques [8].

Our findings are consistent with previous studies demonstrating that a higher NLR and MLR are associated with increased cardiovascular risk and adverse outcomes [9,10]. The SIRI, which incorporates neutrophils, monocytes, and lymphocytes, may provide a more comprehensive assessment of systemic inflammation. An elevated SIRI has been linked to poor prognosis in various cardiovascular conditions [11].

Moreover, the lack of strong correlations between inflammatory indices and lipid biomarkers suggests that inflammation and lipid metabolism may contribute to atherosclerosis through independent but possibly synergistic pathways. This underscores the multifactorial nature of atherosclerosis and the importance of addressing both inflammation and dyslipidemia in prevention and treatment strategies.

### 4.2. Lipid Biomarkers and Their Clinical Significance

Lipid abnormalities are central to the pathogenesis of atherosclerosis. Elevated levels of TC and LDL-C were observed in the main group, while HDL-C levels were lower. LDL-C is well known for its atherogenic potential, contributing to plaque formation when oxidized and taken up by macrophages [6]. HDL-C exerts protective effects by facilitating reverse cholesterol transport and possessing anti-inflammatory properties [12].

Lp(a) levels were significantly higher in patients with atherosclerosis. Lp(a) is structurally similar to LDL but contains an additional apolipoprotein(a) component, which imparts pro-atherogenic and pro-thrombotic properties [13]. Elevated Lp(a) is an independent risk factor for cardiovascular disease, and its levels are largely genetically determined [14,15]. Therapeutic options to lower Lp(a) are limited, but novel agents are under investigation [16].

ANGPTL3 emerged as a key lipid biomarker in our study. It inhibits lipoprotein lipase and endothelial lipase, leading to increased plasma levels of triglycerides and LDL-C [17]. The positive correlation between ANGPTL3 and PLR suggests an interplay between lipid metabolism and platelet-driven inflammation. Recent studies have identified ANGPTL3 as a potential therapeutic target, with monoclonal antibodies and antisense oligonucleotides showing promise in lowering lipid levels [18].

The identification of optimal thresholds for lipid biomarkers provides practical cut-off points for clinical use. For instance, an LDL-C threshold of 1.97 mmol/L showed high sensitivity and could be used to identify individuals who may benefit from early lipid-lowering interventions.

### 4.3. Machine Learning Models and Predictive Insights

The application of machine learning models, such as Random Forest and Gradient Boosting classifiers, provided valuable insights into the predictive capabilities of various biomarkers. SIRI and ANGPTL3 were consistently identified as top predictors, highlighting their importance in early atherosclerosis detection.

While LDL-C is widely recognized as a major risk factor for atherosclerosis, our feature importance analysis found NLR to be a stronger predictor of early atherosclerosis in this cohort. This finding underscores the central role of inflammation in atherogenesis, particularly in younger patients or those without advanced lipid abnormalities. The higher ranking of NLR suggests that inflammatory mechanisms, as reflected by the ratio of neutrophils to lymphocytes, may contribute significantly to early plaque formation. Although LDL-C remains important, this result emphasizes the need to consider both lipid management and inflammation control in early atherosclerosis prevention strategies.

The high AUC values (>0.80) indicate excellent model performance. These models can handle complex, non-linear relationships and interactions between variables, making them suitable for biomedical data [19]. Incorporating machine learning approaches in clinical settings could enhance risk stratification and personalized medicine.

However, it is important to consider that machine learning models require validation in larger, independent cohorts before being implemented in clinical practice. Additionally, the interpretability of such models is crucial for clinician acceptance, and efforts should be made to develop explainable AI tools.

### 4.4. Clustering Analysis and Patient Stratification

Clustering analysis revealed two distinct patient subgroups:

- Inflammation-dominant cluster: Patients with higher inflammatory indices may benefit from anti-inflammatory therapies. This aligns with studies like the CANTOS trial, where targeting inflammation with canakinumab reduced cardiovascular events [2].
- Lipid metabolism cluster: Patients with elevated ANGPTL3 and lipid abnormalities may benefit from lipid-lowering therapies targeting specific pathways. Novel agents targeting ANGPTL3 could offer therapeutic benefits [18].

Understanding these subgroups supports the move towards personalized medicine, tailoring interventions based on individual risk profiles and underlying pathophysiological mechanisms. For example, patients in the inflammation-dominant cluster might be prioritized for interventions targeting inflammation, while those in the lipid metabolism cluster might benefit more from aggressive lipid-lowering strategies.

### 4.5. Clinical Implications and Future Directions

The identification of optimal thresholds for SIRI, ANGPTL3, and lipid parameters provides actionable insights for clinical practice. Implementing these biomarkers in routine practice could improve early detection of atherosclerosis in young adults and guide preventive strategies.

### 4.6. Strengths and Limitations

This study has several strengths, including a comprehensive analysis of both inflammatory and lipid biomarkers and the use of advanced statistical and machine learning techniques. However, there are limitations to consider. The cross-sectional design limits the ability to establish causality. The sample size is relatively small, and participants were from a single center, which may affect the generalizability of the results. Potential confounders such as diet, physical activity, and socioeconomic factors were not accounted for and could influence biomarker levels. Additionally, we did not assess other emerging biomarkers and genetic factors that may contribute to atherosclerosis risk.

## 5. Limitations

- Sample size and diversity: the relatively small and homogeneous sample may limit the applicability of the findings to broader populations.
- Cross-sectional nature: the study design does not allow for causal inferences or assessment of temporal relationships.
- Unmeasured confounders: factors like diet, exercise, and genetic predispositions were not controlled for, which could impact the results.
- Single-center study: findings may not reflect variations in other settings or regions.
- Biomarker variability: biological variability and measurement errors could affect biomarker levels.

## 6. Main Findings

In this study, we conducted a comprehensive analysis of the relationships between inflammatory indices, lipid biomarkers, and the presence of atherosclerosis in young patients aged 18 to 55 years.

- Correlation analysis revealed significant associations among biomarkers. Lp(a) showed a moderate correlation with ApoB (r = 0.43, *p* < 0.01), indicating a link between Lp(a) and ApoB levels. ANGPTL3 exhibited a positive correlation with the PLR (r = 0.38, *p* < 0.01), suggesting possible interactions between angiogenic factors and platelet-mediated inflammatory processes.
- Group comparisons using the Mann–Whitney U test demonstrated significant differences between the main and control groups. Patients with atherosclerosis had elevated inflammatory indices such as SIRI, NLR, MLR, and SII (*p* < 0.01). Additionally, the following lipid profile abnormalities were observed: increased levels of TC and LDL-C, and decreased levels of HDL-C (*p* < 0.05). Lp(a) levels were significantly higher in the main group (*p* < 0.001).
- Logistic regression indicated that SIRI and ANGPTL3 were the most significant predictors of atherosclerosis presence. The model demonstrated high accuracy (77%), high sensitivity (93%), and moderate specificity (72%).
- Feature importance analysis using Random Forest and Gradient Boosting models confirmed the significance of SIRI and ANGPTL3, as well as traditional lipid biomarkers (LDL-C, TC, HDL-C) in predicting atherosclerosis. This highlights the importance of both inflammatory and lipid factors in disease development.
- ROC analysis showed the high discriminatory ability of the models, with an AUC of 0.84 for Random Forest and 0.85 for Gradient Boosting, indicating their effectiveness in distinguishing between patients with and without atherosclerosis.
- Clustering analysis identified two subgroups of patients: one with predominant inflammatory markers and another with pronounced lipid metabolism disturbances. This underscores the heterogeneity of atherosclerosis pathophysiological mechanisms and the need for personalized approaches to treatment and prevention.

## 7. Conclusions

This study underscores the integral roles of systemic inflammation and lipid abnormalities in the early development of atherosclerosis among young adults. The SIRI and ANGPTL3 emerged as potent biomarkers for predicting disease presence, with machine learning models demonstrating high predictive accuracy. The identification of distinct patient subgroups highlights the potential for personalized medicine approaches. Further research with larger, diverse cohorts and a longitudinal follow-up is warranted to validate these findings and explore targeted interventions.

## Figures and Tables

**Table 1:** Clinical and demographic characteristics of patients.

	Main Group (*n* = 62)	Control Group (*n* = 27)
Age, Me (years)	49	47.7
Current Smokers, %	64.5	46.2
Diabetus Miletus, %	30.6	0
Arterial Hypertension, %	82.3	29.6
BMI, Me (kg/m^2^)	28.7	27.9
Statin use, %	66.1	7.4
Aspirin use, %	58.1	3.7
P2Y12 use, %	38.7	0
Family history of early cardiac events, %	30.6	37
Creatinine, Me (mmol/L)	80.4	77.8
TC, Me (mmol/L)	4.62	5.76
LDL, Me (mmol/L)	2.87	3.42
HDL, Me (mmol/L)	0.99	1.28
TG, Me (mmol/L)	2.55	2.63

**Table 2:** Comparison of key variables between main and control groups.

Variable	Main Group (Median)	Control Group (Median)	*p*-Value
NLR	2.5	1.8	<0.01
MLR	0.3	0.2	<0.01
SII	Elevated	Lower	<0.01
SIRI	Elevated	Lower	<0.01
TC (mmol/L)	6.2	5.4	<0.05
LDL-C (mmol/L)	3.8	3.0	<0.05
HDL-C (mmol/L)	1.1	1.4	<0.05
Lp(a) (mg/dL)	30	10	<0.001

**Table 3:** Optimal thresholds for key biomarkers with corresponding sensitivity and specificity.

Biomarker	Threshold	Sensitivity	Specificity
SIRI	1.1	82%	75%
ANGPTL3	55,416 pg/mL	80%	78%
LDL-C	1.97 mmol/L	85%	70%
TC	7.64 mmol/L	75%	80%
HDL-C	2.74 mmol/L	78%	72%

## Data Availability

The primary data analysed in this study are not publicly available due to the policy of access to clinical data of the Scientific Research Institute—Regional Clinical Hospital No. 1 named after prof. S.V. Ochapovsky. However, some parameters that do not contain personal information can be provided by the corresponding author upon reasonable request.
